# Emerging Entities in Vascularized Composite Allotransplantation: A New Layer to Ongoing Challenges

**DOI:** 10.3389/ti.2025.15420

**Published:** 2025-10-17

**Authors:** Haizam Oubari, Yanis Berkane, Curtis L. Cetrulo, Alexandre G. Lellouch

**Affiliations:** ^1^ Plastic Surgery Department, Massachusetts General Hospital Center for Transplantation Sciences, Charlestown, MA, United States; ^2^ Plastic Surgery Department, Cedars-Sinai Medical Center, Los Angeles, CA, United States

**Keywords:** vascularized composite allotransplantation, VCA, whole eye transplantation, WET, swine models

We read with great interest the recent article by Leonard Knoedler et al., titled *“Experimental Swine Models for Vascularized Composite Allotransplantation and Immunosuppression: A Systematic Review and Case Report of a Novel Heterotopic Hemifacial Swine Model”*, which provides a thorough overview of the current strategies for VCA studies in swine. We commend the authors for their comprehensive synthesis of recent advancements in this evolving field and their description of a novel heterotopic partial face transplant model in this species. Experimental VCA models for immunological studies must incorporate the essential components of VCA, and the innovative hemifacial model aligns well with that requirement. Of particular interest, the design conveniently includes mucosal tissue. It presents anatomical features that allow for the integration of vascularized bone marrow, an element shown to be relevant in tolerance induction studies through the establishment of mixed hematopoietic chimerism [1]. Furthermore, porcine models are especially valuable: while nonhuman primates remain a cornerstone for certain translational endpoints, their use is constrained by cost, ethical considerations, and regulatory restrictions. They are therefore most critical at the final stage of translational research, typically after compiling strong data from small animal and swine studies. Pigs offer anatomical and physiological similarities to humans, facilitating surgical refinement, preservation protocol optimization, and immunologic studies. Importantly, experimental studies in whole-eye transplantation must be ethically justified by their potential to advance vision restoration, and should adhere to established animal welfare frameworks [2] as well as recent field-specific ethical analyses [3]. At the same time, heterotopic replantation studies, although not directly assessing visual restoration, can provide critical insights into *ex vivo* preservation strategies and graft viability, which are indispensable steps toward making functional WET a clinical reality, while also informing the design of future orthotopic models that impose a higher experimental burden on the recipient animal.

Among the diverse spectrum of VCAs, a new entity has emerged: whole-eye transplantation (WET). This groundbreaking procedure was first performed by Rodriguez et al. at NYU [4, 5] as part of a face VCA, aiming for morphological restoration in a patient who had sustained a severe facial injury with loss of the left eye. This groundbreaking achievement has renewed hope for patients with ocular blindness by demonstrating the technical feasibility of whole-eye transplantation, albeit without vision restoration to date. It has also underscored the need for extensive preclinical research to optimize key aspects such as graft preservation, nerve coaptation, and immunosuppressive strategies. In parallel, it has stimulated renewed interest in WET research, building on earlier work already undertaken in several animal models, including orthotopic replantation experiments in rodents [6], as well as human anatomical studies [7–9]. The porcine whole eye vascularized composite allotransplant model has been described [10, 11] and presents several distinct anatomical advantages. It includes the eyeball, palpébra, lacrimal gland, and intraorbital content. Notably, the absence of a lateral orbital wall [12] allows for a vascular configuration that is particularly favorable for procurement and experimental manipulation. The facial vein, originating from the frontal vein and forming part of the external jugular vein system, shares a communicating branch with the ophthalmic vein. On the arterial side, the ophthalmic artery maintains a direct communicating branch with the external carotid system, allowing the arterial pedicle of the WET to be dissected from the ophthalmic artery proximally to the external and common carotid arteries in the neck. This feature facilitates *ex vivo* experiments and transplantation studies, enabling WET procurement solely via the external carotid artery and jugular veins without intracranial dissection (Figure 1). Our group has recently refined and adapted this porcine model for *ex vivo* machine perfusion studies [13] and confirmed that these anatomical features are consistent across different pig strains, including Yucatan, Yorkshire, and common commercial breeds (unpublished data). Notably, the WET unit can also be combined with other facial components into chimeric composite flaps, including the ear and additional facial subunits.

**FIGURE 1:**
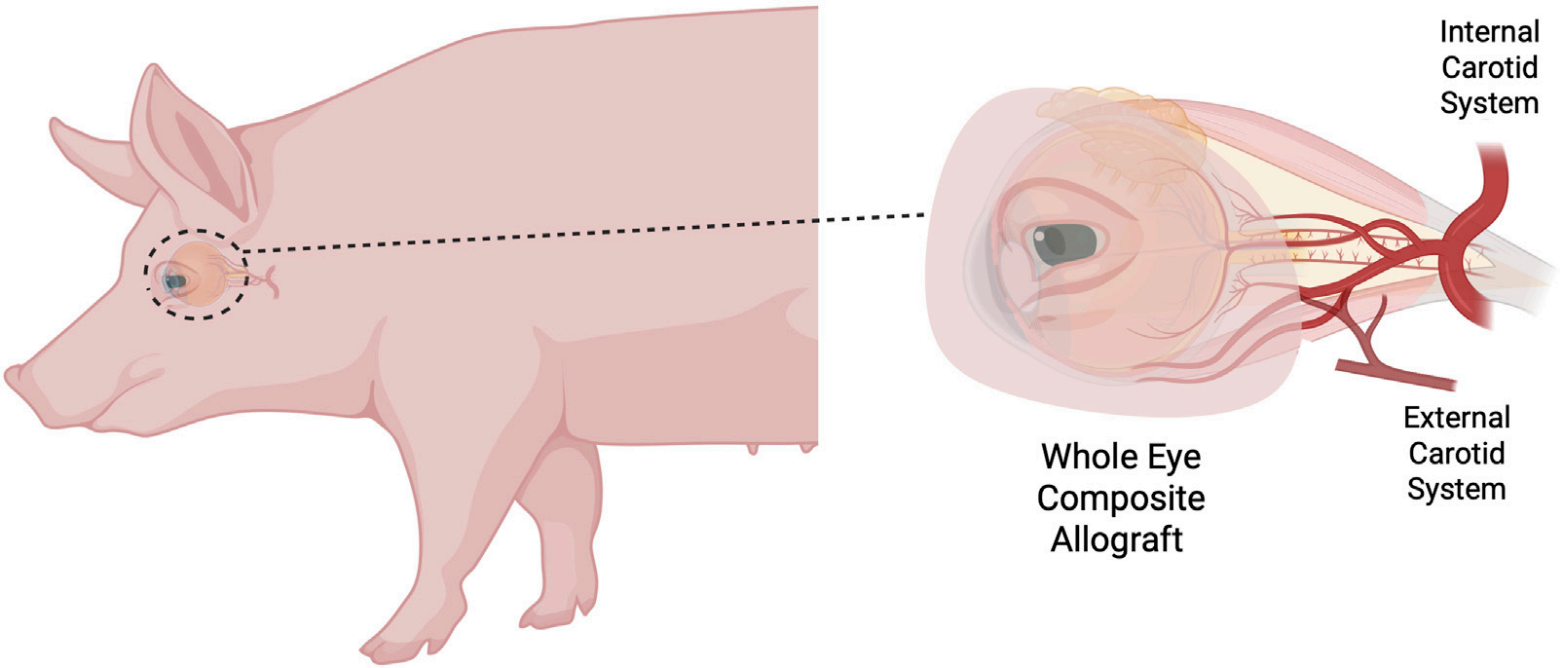
Whole eye transplantation model in the pig.

Non–skin-bearing VCA models, such as uterine [14] and laryngeal [15] transplantation in swine, have also been developed, offering valuable insights into surgical training, preservation strategies, and immunosuppression. However, these models lie beyond the scope of the present discussion. As WET represents an even more complex and sensitive category of VCA that has only recently emerged in clinical practice, it fully fits within the VCA domain. While it holds remarkable translational promise, substantial work remains to address its unique anatomical, immunological, and neurophysiological challenges.

In conclusion, incorporating WET models into future VCA studies will be essential to tackle key hurdles, including immunological compatibility, preservation strategies, and restoration of function. Fostering close collaboration among microsurgeons, transplant immunologists, and neuroscientists will be critical to accelerating the translation of experimental advances into effective clinical protocols for WET, and VCA more broadly.

## Data Availability

The original contributions presented in the study are included in the article/supplementary material, further inquiries can be directed to the corresponding authors.

## References

[1] Barth RN Rodriguez ED Mundinger GS Nam AJ Ha JS Hui-Chou H Vascularized Bone Marrow-Based Immunosuppression Inhibits Rejection of Vascularized Composite Allografts in Nonhuman Primates. Am J Transpl (2011) 11(7):1407–16. 10.1111/j.1600-6143.2011.03551.x 21668624

[2] National Research Council (US). In: Committee for the Update of the Guide for the Care and Use of Laboratory Animals. Guide for the Care and Use of Laboratory Animals. 8th ed. Washington (DC): National Academies Press (2011). Available online at: https://nap.nationalacademies.org/read/12910/chapter/1 (Accessed September 15, 2025).

[3] Caplan A. Reopening the “Window to the Soul”? The Ethics of Eye Transplantation now and in the Future. Am J Bioeth (2024) 24(5):6–7. 10.1080/15265161.2024.2333218 38635437

[4] Nowogrodzki J. World's First Whole-Eye Transplant: The Innovations that Made It Possible. Nature (2024) 633(8030):500–1. 10.1038/d41586-024-02906-4 39251795

[5] Lico M Hanley K Shah A Chinta S Ceradini DJ Rodriguez ED. Swallowing Function After Pioneering Partial Face and Whole Eye Transplant: Clinical Insights. Am J Speech Lang Pathol (2025) 34(4):1921–30. 10.1044/2025_AJSLP-24-00364 40403408

[6] Li Y Komatsu C He L Miller MR Noori J van der Merwe Y Surgical Techniques and Outcome Assessment of a Novel Vascularized Orthotopic Rodent Whole Eye Transplantation Model. PLoS One (2025) 20(5):e0311392. 10.1371/journal.pone.0311392 40408444 PMC12101781

[7] Siemionow M Bozkurt M Zor F Kulahci Y Uygur S al OC A New Composite Eyeball-Periorbital Transplantation Model in Humans: An Anatomical Study in Preparation for Eyeball Transplantation. Plast Reconstr Surg (2018) 141(4):1011–8. 10.1097/PRS.0000000000004250 29595735

[8] Brydges HT Onuh OC Chaya BF Tran DL Cassidy MF Dedania VS Combined Face and Whole Eye Transplantation: Cadaveric Rehearsals and Feasibility Assessment. Plast Reconstr Surg Glob Open (2023) 11(11):e5409. 10.1097/GOX.0000000000005409 38025647 PMC10653600

[9] Davidson EH Wang EW Yu JY Fernandez-Miranda JC Wang DJ Richards N Total Human Eye Allotransplantation: Developing Surgical Protocols for Donor and Recipient Procedures. Plast Reconstr Surg (2016) 138(6):1297–308. 10.1097/PRS.0000000000002821 27879599 PMC5457795

[10] Bravo MG Granoff MD Johnson AR Lee BT. Development of a New Large-Animal Model for Composite Face and Whole-Eye Transplantation: A Novel Application for Anatomical Mapping Using Indocyanine Green and Liquid Latex. Plast Reconstr Surg (2020) 145(1):67e–75e. 10.1097/PRS.0000000000006322 31577655

[11] Rousou C Hoogenboom P van Overdam KA Storm G Dorrestijn J Mastrobattista E. A Technical Protocol for an Experimental Ex Vivo Model Using Arterially Perfused Porcine Eyes. Exp Eye Res (2019) 181:171–7. 10.1016/j.exer.2019.02.003 30735657

[12] Kyllar M Štembírek J Danek Z Hodan R Stránský J Machoň V A Porcine Model: Surgical Anatomy of the Orbit for Maxillofacial Surgery. Lab Anim (2016) 50(2):125–36. 10.1177/0023677215577923 25925960

[13] Oubari H Cabanel L Van Dieren L Berkane Y Randolph MA Uygun K 32. Preservation of a Whole Eye Transplant by Machine Perfusion: A First Study on a Porcine Model. Transplantation (2025) 109(6S2):20. 10.1097/01.tp.0001123876.23176.a5 PMC12333806 40785850

[14] Cabanel L Oubari H Dion L Lavoué V Randolph MA Cetrulo CL Establishing a Swine Model to Study Uterus Dynamic Preservation and Transplantation. J Vis Exp (2024) 20:214. 10.3791/67357 39760401 PMC12066085

[15] Henderson D Knoedler L Niederegger T Fenske J Mathieu O Hundeshagen G What are the Functional Outcomes of Total Laryngeal Transplantation? A Systematic Review of Preclinical and Clinical Studies. Front Immunol (2025) 16:1631525. 10.3389/fimmu.2025.1631525 40677707 PMC12267005

